# Meleagrin Isolated from the Red Sea Fungus *Penicillium chrysogenum* Protects against Bleomycin-Induced Pulmonary Fibrosis in Mice

**DOI:** 10.3390/biomedicines10051164

**Published:** 2022-05-18

**Authors:** Sameh S. Elhady, Marwa S. Goda, Eman T. Mehanna, Mahmoud A. Elfaky, Abdulrahman E. Koshak, Ahmad O. Noor, Hanin A. Bogari, Rania T. Malatani, Reda F. A. Abdelhameed, Alaa S. Wahba

**Affiliations:** 1 Department of Natural Products, Faculty of Pharmacy, King Abdulaziz University, Jeddah 21589, Saudi Arabia; melfaky@kau.edu.sa (M.A.E.); aekoshak@kau.edu.sa (A.E.K.); 2 Department of Pharmacognosy, Faculty of Pharmacy, Suez Canal University, Ismailia 41522, Egypt; marwa_saeed@pharm.suez.edu.eg; 3 Department of Biochemistry, Faculty of Pharmacy, Suez Canal University, Ismailia 41522, Egypt; alaa.samir@pharm.suez.edu.eg; 4 Centre for Artificial Intelligence in Precision Medicines, King Abdulaziz University, Jeddah 21589, Saudi Arabia; 5 Department of Pharmacy Practice, Faculty of Pharmacy, King Abdulaziz University, Jeddah 21589, Saudi Arabia; aonoor@kau.edu.sa (A.O.N.); hbogari@kau.edu.sa (H.A.B.); rmalatani@kau.edu.sa (R.T.M.); 6 Department of Pharmacognosy, Faculty of Pharmacy, Galala University, New Galala 43713, Egypt; reda.fouad@gu.edu.eg

**Keywords:** *Penicillium chrysogenum*, meleagrin, bleomycin, pulmonary fibrosis, oxidative stress, inflammation, apoptosis, drug discovery, industries development

## Abstract

The Red Sea marine fungus *Penicillium chrysogenum* (Family: Ascomycota) comprises a panel of chemically diverse natural metabolites. A meleagrin alkaloid was isolated from deep-sediment-derived *P. chrysogenum* Strain S003 and has been reported to exert antibacterial and cytotoxic activities. The present study aimed to explore the therapeutic potential of meleagrin on pulmonary fibrosis. Lung fibrosis was induced in mice by a single intratracheal instillation of 2.5 mg/kg bleomycin. Mice were given 5 mg/kg meleagrin daily either for 3 weeks after bleomycin administration in the treatment group or 2 weeks before and 3 weeks after bleomycin administration in the protection group. Bleomycin triggered excessive ROS production, inflammatory infiltration, collagen overproduction and fibrosis. Bleomycin-induced pulmonary fibrosis was attenuated by meleagrin. Meleagrin was noted to restore the oxidant–antioxidant balance, as evidenced by lower MDA contents and higher levels of SOD and catalase activities and GSH content compared to the bleomycin group. Meleagrin also activated the Nrf2/HO-1 antioxidant signaling pathway and inhibited TLR4 and NF-κB gene expression, with a subsequent decreased release of pro-inflammatory cytokines (TNF-α, IL-6 and IFN-γ). Additionally, meleagrin inhibited bleomycin-induced apoptosis by abating the activities of pro-apoptotic proteins Bax and caspase-3 while elevating Bcl2. Furthermore, it suppressed the gene expression of α-SMA, TGF-β1, Smad-2, type I collagen and MMP-9, with a concomitant decrease in the protein levels of TGF-β1, α-SMA, phosphorylated Smad-2, MMP-9, elastin and fibronectin. This study revealed that meleagrin’s protective effects against bleomycin-induced pulmonary fibrosis are attributed to its antioxidant, anti-inflammatory, anti-apoptotic and antifibrotic properties. Notably, the use of meleagrin as a protective agent against bleomycin-induced lung fibrosis was more efficient than its use as a treatment agent.

## 1. Introduction

Pulmonary fibrosis is the most common interstitial lung disease with a high mortality and morbidity due to its progressive and irreversible nature. Pulmonary fibrosis is characterized by reactive oxygen species (ROS) generation, inflammatory cells infiltration, fibroblast proliferation and differentiation into myofibroblasts, eventually leading to extracellular matrix protein deposition into the lung parenchyma irreversibly destroying the lung architecture [1,2].

Triggering factors, such as environmental toxins (asbestos, silica and some gases), infections, acute pulmonary injury or inflammation, connective tissue disorder, radiation therapy and chemotherapeutic agents (e.g., bleomycin), have been demonstrated to attribute to the persistent progress of pulmonary fibrosis [2]. Bleomycin-induced pulmonary fibrosis is the most widely used rodent model resembling human lung fibrosis. In response to oxidative stress, pulmonary cells release pro-inflammatory cytokines (tumor necrosis factor-α (TNF-α), interleukin (IL) 1β and IL-8), inducing leukocyte infiltration and nuclear factor kappa B (NF-κB) activation [3]. Transforming growth factor-beta 1 (TGF-β1) is a crucial cytokine associated with the induction and development of pulmonary injury. TGF-β binding to its receptors could phosphorylate mothers against the decapentaplegic homolog (Smad) signaling pathway and induce collagen synthesis by stimulation of fibroblast proliferation. Furthermore, mitochondrial injury and epithelial apoptosis have been shown to be critically involved in the progression of lung fibrosis [4].

Anti-inflammatory drugs have a therapeutic potential on early pulmonary inflammation and subsequent scarring; however, their use does not halt fibrosis progression. Therefore, lung transplantation is considered a more effective therapeutic option [2]. Recently, pirfenidone and nintedanib have been approved by the U.S. food and drug administration as a treatment for the clinical course of pulmonary fibrosis to slow the deterioration of lung function [5]. However, there is still no cure for pulmonary fibrosis, which necessitates the need for more effective therapies [2].

The marine environment possesses unique biological and chemical characters that play a vital role in the discovery of many drug leads for the treatment of various diseases. Marine fungi have been widely distributed and diversified taxonomically, and it is estimated that there are approximately greater than 10,000 marine fungal species. Most marine fungi are pertaining to Ascomycota and Basidiomycota families [6]. They are considered as a wealth of natural products that have many pharmacological effects. These marine bio medicinal agents have been isolated, structurally elucidated and biologically evaluated [7].

The genus *Penicillium* is an ascomycetous fungus that is of major importance in food and drug production. *Penicillium* species are a rich treasury of various natural metabolites of pharmaceutical and medicinal values [8]. Among them, Penicillium sp. showed significant antibacterial [9], anti-inflammatory and α-glucosidase effects [10], in addition to a potent cytotoxic activity against cervical cancer (HeLa), hepatic cancer (HepG2), breast cancer (MCF-7) and colon cancer (HCT115) cell lines [9].

Industrially, the fungus *Penicillium chrysogenum* is used for penicillin production [11]. *P*. *chrysogenum* has been found to biosynthesize different classes of natural metabolites, such as polyketides, terpenoids, alkaloids, cerebrosides [12] and sterols [13]. These natural metabolites were isolated and identified as deoxyartemisinin [14], trichodimerol [15], berkeleydione and berkeleytrione terpenoids [16], sorbicillinoid alkaloids, sorbicillactones [17], xanthohumol and its derivatives [18], xanthocillin X polyketide [19], roquefortine C alkaloid, meleagrin alkaloid [11], chrysogeside cerebrosides, chrysogedone alkaloids [12], penicilloside cerebrosides [20], ergosterol, epidioxyergosterol and kojic acid [13].

Meleagrin alkaloid was reported to have antibacterial effect against Gram-positive bacteria through the inhibition of bacterial enoyl-acyl carrier protein reductase, which is essential for bacterial cell growth, and does not interfere with mammalian fatty acid synthesis [21]. It also exhibited a significant cytotoxic activity against different types of human cancer cell lines, such as the breast cancer cell line (MCF-7), lung cancer cell line (A-549), human leukemia cell line (HL-60), cervical cell line (BEL-7402) and T-lymphoblast cell line (MOLT-4) [8].

Here, the isolation of meleagrin from the Red Sea deep-sediment fungus *P. chrysogenum* strain S003 is handled as part of ongoing efforts to investigate the protective effect of meleagrin against bleomycin-induced lung toxicity in a mouse model.

## 2. Materials and Methods

### 2.1. Collection and Isolation of Marine P. chrysogenum

**Figure 1:**
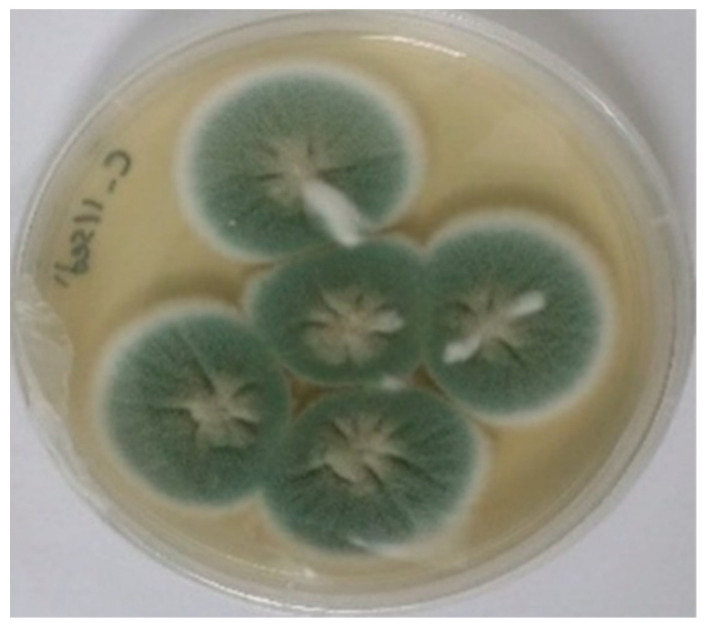
A morphological photo of *P. chrysogenum* strain S003 [22].

The deep-sea sediment was collected from the Saudia Arabian Red Sea. As mentioned previously [22], the deep-sea sediment was homogenized with sterile water and serially diluted under aseptic conditions. An amount of 90 µL was cultured on different culture media, Czapek–Dox yeast agar medium, malt extract agar medium and Sabouraud dextrose agar medium. These media were supplemented with chloramphenicol antibiotic and sodium chloride. After an incubation period of 2 weeks, a series of purification steps were carried out to obtain pure fungal strain (Figure 1). The fungal strain was identified morphologically as well as genotypically based on a previously described method [22]

### 2.2. Fermentation Method and Extraction Process

As described before [13], the fungus *P. chrysogenum* strain S003 was cultured on Czapek–Dox yeast liquid culture medium at ambient temperature. After an incubation period of 30 days, the broth media were filtered. On the one hand, the liquid broth media were partitioned with ethyl acetate three times to extract active metabolites. The combined ethyl acetate (EtOAc) extract was concentrated under vacuum to afford 1.4 g dry extract. On the other hand, the mycelia were also extracted with methanol.

### 2.3. Isolation and Purification of Pure Meleagrin

The EtOAc extract was chromatographed over Sephadex LH-20 column and eluted with CHCl3: MeOH (1:1) to yield 10 sub-fractions (EtOAc-1 to EtOAc-10) based on TLC analysis. Fraction EtOAc-5 (433 mg) was subjected to fractionation over Sephadex LH-20 column and eluted with CHCl3: MeOH (1:1) to yield 4 sub-fractions (EtOAc-5-1′ to EtOAc-5-4′). Fraction EtOAc-5-2′ (213 mg) was finally purified over silica gel column using n-hexane: EtOAc (9:1~1:9) gradient elution to give pale-yellow crystal meleagrin (120 mg).

### 2.4. Investigation of Meleagrin’s Bilogical Action in a Mouce Model of Bleomycin-Induced Lung Fibrosis

#### 2.4.1. Drugs and Treatment Preparation

Bleomycin (Bleocel 15) was purchased from Celon, Telangana, India. Pulmonary fibrosis was induced by single endotracheal administration of sublethal dose of bleomycin (2.5 U/kg) [23] dissolved in saline (0.9% NaCl), given by the transoral route with sterile disposable plastic loading pipette tips under ketamine anesthesia. Bleomycin was freshly prepared at the day of induction. Meleagrin was administered in a dose of 5 mg/kg/day dissolved in (1% dimethyl sulfoxide [DMSO] in saline solution) and was freshly prepared twice per week and stored at 4 °C. The concentration was adjusted so that each animal received bleomycin and meleagrin doses in 25 µL saline and 100 µL 1% dimethyl sulfoxide/kg body weight, respectively, taking into account changes in body weight through the experiment.

#### 2.4.2. Experimental Design

Forty male Swiss Albino mice, aged from 6–7 weeks, were purchased from the Faculty of Veterinary Medicine, Suez Canal University (Ismailia, Egypt) and acclimatized for 1 week. The mice were kept in stainless steel cages at controlled temperature (25 ± 2 °C), and had free access to food and water with 12 h light/dark cycle. Only male mice were used to overcome sexual dimorphism associated with bleomycin-induced lung fibrosis [24]. The animals were randomly divided into the following five groups (*n* = 8 per group): vehicle control group (treated with agents’ vehicles; mice received 25 µL intratracheal saline alone followed by daily oral administration of 100 µL 1% DMSO/saline for 3 weeks), meleagrin group (mice received 25 µL intratracheal saline alone followed by daily oral administration of 5 mg/kg meleagrin dissolved in 100 µL 1% DMSO/saline for 3 weeks), bleomycin group (mice received 25 µL single intratracheal dose of 2.5 U/kg bleomycin in saline, then 100 µL 1% DMSO/saline, orally, daily for 3 weeks), bleomycin + meleagrin group (mice received 25 µL single intratracheal dose of 2.5 U/kg bleomycin in saline, then treated with 5 mg/kg meleagrin dissolved in 100 µL 1% DMSO/saline, orally, daily for 3 weeks after bleomycin administration) and meleagrin + bleomycin group (25 µL single intratracheal dose of 2.5 U/kg bleomycin in saline, and treated with 5 mg/kg meleagrin dissolved in 100 µL 1% DMSO/saline, orally, daily for 2 weeks before and 3 weeks after bleomycin administration). Experimental design was approved by the Research Ethics Committee of Faculty of Pharmacy, Suez Canal University (202103RA3). Animal handling was conducted following Guide for the Care and Use of Laboratory Animals (8th edition, National Academies Press).

At the end of the experiment, mice were decapitated. Lung tissues were harvested and rinsed in ice-cold saline. The left lobes from all of the lungs were divided to two portions, stored immediately at −80 °C and used for preparation of lung homogenate. The first portion was weighed and prepared as 10% homogenates in 0.9% saline, and centrifuged, and the resulting supernatant was collected and used for determination of oxidative stress markers and enzyme linked immunosorbent assay (ELISA) measurements. The other portion’s homogenate was prepared in Qiazol reagent for PCR analysis. The right lobes were fixed immediately in 10% formalin buffered saline, routinely processed to be embedded in paraffin wax, cut into 5 μm sections and stained for immunohistochemical, histopathological and morphometrical analysis.

#### 2.4.3. Determination of Oxidative Stress Biomarkers

Oxidative stress status was assessed by estimation of malondialdehyde (MDA) and reduced glutathione (GSH) contents, and catalase and superoxide dismutase (SOD) activities in lung homogenate using Bio diagnostic colorimetric assay kits (Dokki, Giza, Egypt, Cat. No. MD 2529 for MDA, GR 2511 for GSH, CA 2517 for catalase and SD 2521 for SOD). All procedures were performed in accordance with manufacturer’s instructions.

#### 2.4.4. Quantitative RT-PCR Analysis for Determination of Inflammatory and Fibrotic Biomarkers

The gene expression of nuclear factor erythroid 2–related factor 2 (Nrf2), NF-κB, toll like receptor 4 (TLR4), TNF-α, type I collagen, TGF-β1, α-smooth muscle actin (α-SMA), Smad-2 and matrix metalloproteinase-9 (MMP-9) genes was assessed in lung homogenate using quantitative RT-PCR technique. Extraction of total RNA from lung tissue was performed using miRNeasy Mini kit Isolation kit (Qiagen, Hilden, Germany) following the manufacturers’ instructions. Purity and concentration of the extracted RNA were determined spectrophotometrically at 260/280 nm using the NanoDrop ND-1000 (NanoDrop Tech., Wilmington, DE, USA). The expression level of the assessed genes was analyzed using GoTaq^®^ 1-Step RT-qPCR System (Promega, Madison, WI, USA) with the β-actin used as a reference control. As figured by the manufacturer, reverse transcription and PCR reaction were carried out in one step in 20 μL total volume that consisted of RNA template (4 μL), forward and reverse primers (2 μL each), GoTaq^®^ qPCR master mix (10 μL), GoScript™ RT mix (0.4 μL), CXR reference dye (0.31 μL) and nuclease-free water (3.29 μL). The GenBank accession No., primers’ sequences and annealing temperatures are described in Table 1. Thermal cycling was conducted using the thermocycler StepOne real-time PCR System (Applied Biosystems, Waltham, MA, USA) as follows: 37 °C for 15 min (reverse transcription), 95 °C for 10 min (reverse transcriptase inactivation), followed by 40 cycles at 95 °C for 10 s (denaturation), at the specific annealing temperature for 30 s (annealing) and at 72 °C for 30 s (extension). Then, final extension was carried out in one cycle at 72 °C for 10 min. For all samples, the reaction was run in duplicate, and with a no-template control. The relative expression of assessed genes was determined using the 2^−ΔΔCT^ formula [25].

#### 2.4.5. Measurement of Protein Levels of Inflammatory, Fibrotic and Apoptotic Biomarkers in Lung Homogenate

Heme oxygenase-1 (HO-1) in lung homogenates was assessed using mice HO-1 ELISA kit (Enzo life sciences, Farmingdale, NY, USA, Cat. No. ADI-960-071). Interferon gamma (IFN-γ) and IL-6 levels in the lung homogenate were determined by mice specific ELISA kits (MyBioSource, San Diego, CA, USA, Cat. No. MBS2500105 and MBS2508516, respectively), following the manufacturer protocol.

Levels of TGF-β1, α-SMA, phosphorylated Smad-2 and MMP-9 in the lung homogenate were determined using mouse-specific ELISA kits (MyBioSource, San Diego, CA, Cat. No. MBS164646, MBS267551, MBS269937 and MBS2886011, respectively). The levels of the extracellular matrix proteins elastin and fibronectin were also assessed in the tissue homogenate of the experimental mice by mouse-specific ELISA kits (MyBioSource, San Diego, CA, USA, Cat. No. MBS939028 and MBS494774, respectively).

In addition, the protein levels of the markers of apoptosis caspase-3, B cell lymphoma-2 (Bcl-2) and Bcl-2 associated x (Bax) were determined in the lung homogenate by mouse-specific ELISA kits (MyBioSource, San Diego, CA, USA, Cat. No. MBS733100, MBS2512543 and MBS2607437 respectively) following the instructions of the manufacturer.

#### 2.4.6. Immunohistochemical Examination

Immunohistochemical technique was used to assess caspase-3, Bcl-2 and Bax protein expression in lung samples. Paraffinized lung sections (5 µm thick) were deparaffinized and hydrated in graded ethanol. Antigen retrieval was accomplished by boiling for 5 min in citrate buffer (pH 6.0). Thereafter, endogenous peroxidase was inactivated by 3% H_2_O_2_ in methanol and slides were blocked with 5% bovine serum albumin in phosphate buffered saline (PBS). Then, slides were incubated overnight with diluted anti- caspase-3, anti-Bcl-2 and anti-Bax antibodies in PBS at 4 °C, and then incubated with secondary antibody for 30 min. Detection of bound antibodies was achieved by incubation with avidin–biotin complex (ABC kit, Vector Laboratories, Burlingame, CA, USA) at 37 °C for 45 min. After washing with PBS, the slides were incubated in diaminobenzidine for 10 min and counterstained with Mayer’s hematoxylin. The fraction of immunoreactive area in 5 fields/section was measured (X200) and presented as the percentage of area of immunopositive reaction per field using image analysis software (Leica QWin Plus v3; Leica Microsystems Ltd., Wetzlar, Germany).

#### 2.4.7. Histopathological Examination

The right lobes were fixed in 10% buffered formalin, routinely processed to be embedded in paraffin wax, cut into sections (5 μm thick) and used for histopathological examination. Tissue sections were stained with hematoxylin and eosin (H&E) and Masson’s trichrome (MT), and then examined under light microscope. The structural changes in lung tissue were examined based on the degree of hemorrhage, inflammatory lesions and collagen deposition. Lung fibrosis was scored by a pathologist based on the semiquantitative Ashcroft score [26].

#### 2.4.8. Statistical Analysis

Data were analyzed by Statistical Package for Social Sciences, SPSS (IBM, Armonk, NY, USA), version 21.0 software. Data are expressed as mean ± SD and analyzed using one-way ANOVA followed by Bonferroni’s post hoc (multiple comparison) test. Differences were considered statistically significant at *p* ˂ 0.05.

## 3. Results

### 3.1. Identification of Meleagrin

**Figure 2:**
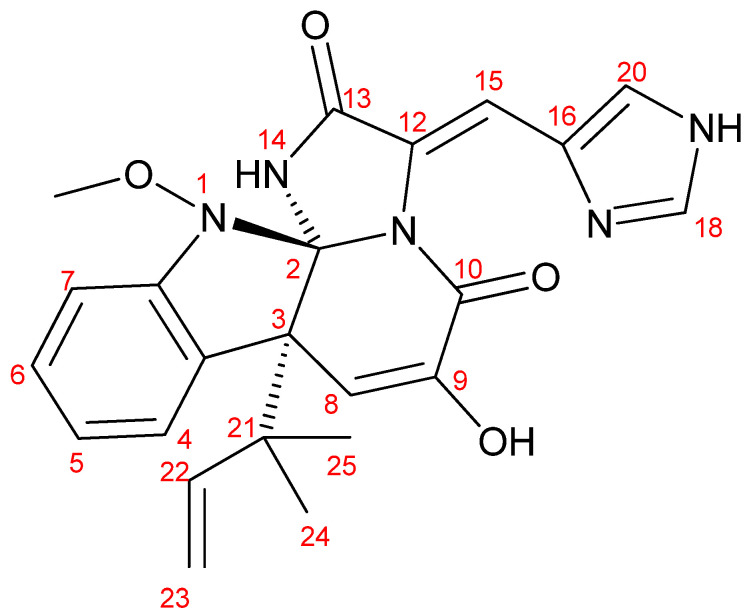
Chemical structure of isolated meleagrin.

Meleagrin, (Figure 2) was obtained as a pale-yellow crystal with a molecular formula of C_23_H_23_N_5_O_4_ and m/z 434 [M + H]^+^. The ^13^C NMR (100 MHz, CDCl_3_:CD_3_OD (1:1)) spectral data: *δ*_C_ 65.4 (1-OCH_3_), 101.6 (C-2), 52.5 (C-3), 126.1 (C-3a), 124.9 (C-4), 122.8 (C-5), 128.5 (C-6), 111.3 (C-7), 146.5 (C-7a), 109.5 (C-8), 141.8 (C-9), 159.2 (C-10), 123.6 (C-12), 167.0 (C-13), 108.5 (C-15), 125.5 (C-16), 134.3 (C-18), 136.8 (C-20), 42.6 (C-21), 141.8 (C-22), 111.3 (C-23) and 29.7 (C-24 and 25). The ^1^H NMR (400 MHz, CDCl_3_) spectral data: *δ*_H_ 3.73 (3H, s, 1-OCH_3_), 7.57 (1H, d, *J* = 7.85, H-4), 7.09 (1H, dt, *J* = 7.55, 0.95, H-5), 7.26 (1H, dt, *J* = 7.8, 0.95, H-6), 6.98 (1H, d, *J* = 7.85, H-7), 5.50 (1H, s, H-8), 8.27 (1H, s, H-15), 7.30 (1H, s, H-18), 7.59 (1H, s, H-20), 6.12 (1H, brs, H-22), 5.05 (1H, d, *J* = 17.1, Ha-23), 5.11 (1H, d, *J* = 9.6, Hb-23), 1.24 (3H, s, CH_3_-24) and 1.34 (3H, s, CH_3_-25). As a result of comparing the ^1^H NMR and ^13^C NMR spectral data with those previously reported in the literature [21,27], the compound was identified to be meleagrin.

### 3.2. In Vivo Investigation of the Effects of Meleagrin in a Model of Bleomycin-Induced Lung Fibrosis in Experimental Mice

#### 3.2.1. Effect of Meleagrin on Markers of Oxidative Stress and Inflammation in the Lung Tissue

**Figure 3:**
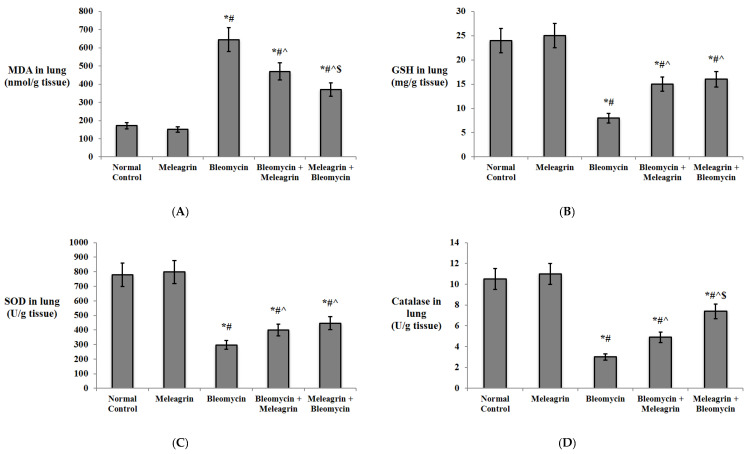
The effect of meleagrin on the levels of markers of oxidative stress (**A**) MDA, (**B**) GSH, (**C**) SOD and (**D**) catalase in the lung tissue of the experimental mice. MDA = malondialdehyde; GSH = reduced glutathione; SOD = superoxide dismutase. Data are expressed as mean ± SD and analyzed using one-way ANOVA followed by Bonferroni’s post hoc test (*n* = 8). * significantly different vs. the normal control group; ^#^ significantly different vs. the meleagrin group; ^ significantly different vs. the bleomycin group. ^$^ significantly different vs. the bleomycin + meleagrin group. Differences were considered significant at *p <* 0.05.

Figure 3 reveals that the administration of meleagrin alone caused no significant alteration in the levels of the oxidative stress markers in the lung tissue compared to the normal control group. On the other hand, bleomycin administration caused a significant increase in the levels of the lipid peroxidation marker MDA and a significant decline in the levels of GSH, SOD and catalase in the lung tissue compared to the normal control group (*p* < 0.05). The administration of meleagrin either as a treatment agent (bleomycin + meleagrin group) or as a protective agent (meleagrin + bleomycin group) caused a significant decrease in the levels of MDA, along with a significant increase in the antioxidant GSH, SOD and catalase in the lung tissue relative to the bleomycin control group. Notably, the use of meleagrin as a protective agent led to significantly lower levels of MDA, but significantly higher levels of catalase when compared with their levels in the lung tissue of the bleomycin + meleagrin group (Figure 3).

**Figure 4:**
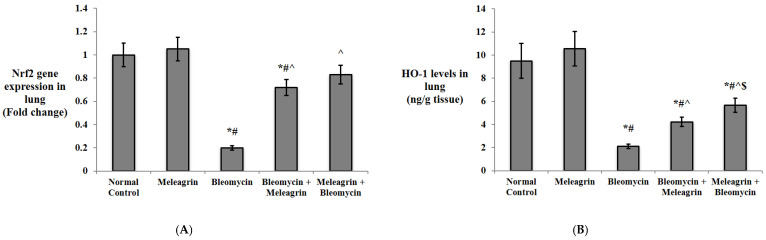
The effect of meleagrin on the Nrf2/HO-1 pathway: (**A**) the gene expression of Nrf2, and (**B**) the levels of HO-1 in the lung tissue of the experimental mice. Nrf2 = nuclear factor-erythroid factor 2-related factor 2; HO-1 = heme oxygenase-1. Data are expressed as mean ± SD and analyzed using one-way ANOVA followed by Bonferroni’s post hoc test (*n* = 8). * significantly different vs. the normal control group; ^#^ significantly different vs. the meleagrin group; ^ significantly different vs. the bleomycin group. ^$^ significantly different vs. the bleomycin + meleagrin group. Differences were considered significant at *p <* 0.05.

The Nrf2/HO-1 pathway was investigated in the current study (Figure 4). Meleagrin alone had no effect on both the gene expression of Nrf2 or the protein level of HO-1 in the lung tissue. The administration of bleomycin significantly downregulated the expression of Nrf-2 and decreased the HO-1 level in the lung tissue in comparison to the normal mice (*p* < 0.05). Mice that received meleagrin either as a treatment (bleomycin + meleagrin) or as a protection (meleagrin + bleomycin) had a significantly higher Nrf2 expression and HO-1 level in the lung tissue compared to the bleomycin control group. Notably, the lung HO-1 level was significantly higher in the meleagrin + bleomycin group relative to the bleomycin + meleagrin group (Figure 4).

**Figure 5:**
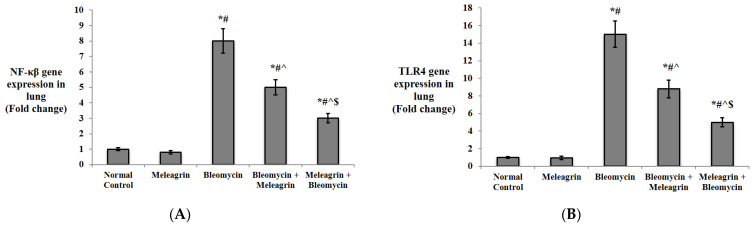

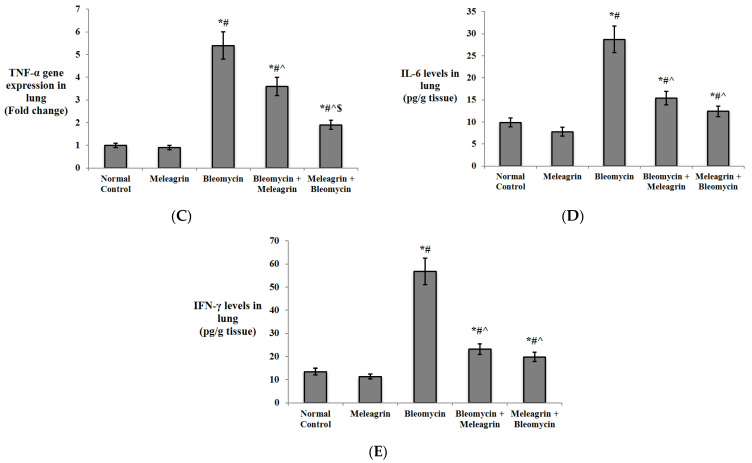
The effect of meleagrin on the inflammatory markers: (**A**) NF-κB gene expression, **(B)** TLR4 gene expression, (**C**) TNF-α gene expression, (**D**) IL-6 levels and (**E**) IFN-γ levels in the lung tissue of the experimental mice. NF-κB = nuclear factor kappa B; TLR4 = toll like receptor 4; TNF-α = tumor necrosis factor-alpha; IL-6 = interleukin-6; IFN-γ = interferon gamma. Data are expressed as mean ± SD and analyzed using one-way ANOVA followed by Bonferroni’s post hoc test (*n* = 8). * significantly different vs. the normal control group; ^#^ significantly different vs. the meleagrin group; ^ significantly different vs. the bleomycin group; ^$^ significantly different vs. the bleomycin + meleagrin group. Differences were considered significant at *p <* 0.05.

Markers of inflammation in the lung tissue of the experimental groups were also detected (Figure 5). Mice that received meleagrin only showed no significant increase in any of the detected inflammatory markers compared to the normal control group. Expectedly, bleomycin caused a significant upregulation of the gene expression of NF-κB, TLR4 and TNF-α, along with a significant increase in IL-6 and IFN-γ protein levels in the lung tissue compared to the meleagrin group (*p* < 0.05). Meleagrin, in both the treatment (bleomycin + meleagrin) and protection (meleagrin + bleomycin) groups, could significantly downregulate NF-κB, TLR4 and TNF-α expression and lower IL-6 and IFN-γ levels in the lung tissue in comparison with the bleomycin control group. Interestingly, the expression of NF-κB, TLR4 and TNF-α in the protected mice (meleagrin + bleomycin) was significantly lower than their expression in the treated mice (bleomycin + meleagrin) (Figure 5).

#### 3.2.2. Effect of Meleagrin on the Expression of Markers of Apoptosis in the Lung Tissue

**Figure 6:**
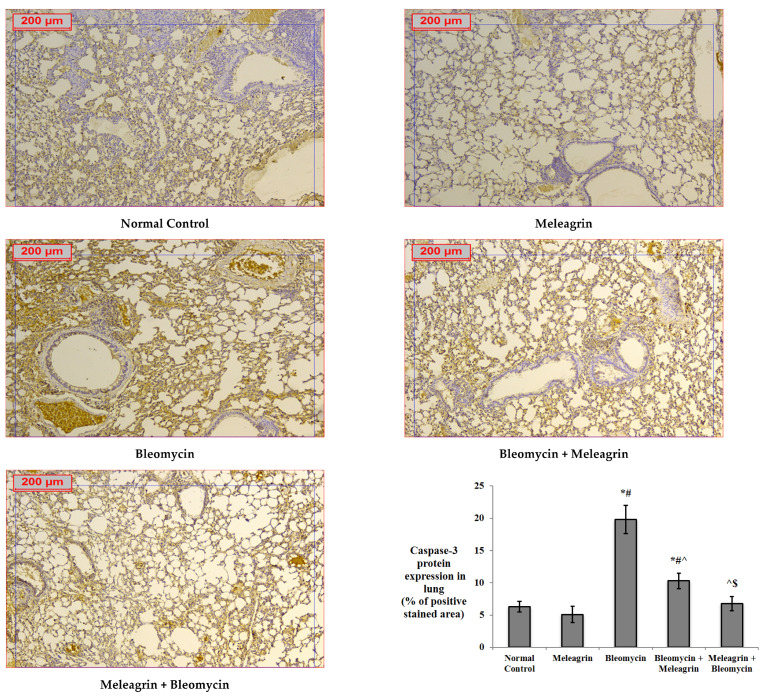
The effect of meleagrin on the protein expression levels of caspase-3 in the lung tissue of the experimental mice determined by immunohistochemistry. Data of the percentage of positive stained area are expressed as mean ± SD and analyzed using one-way ANOVA followed by Bonferroni’s post hoc test (*n* = 8). * significantly different vs. the normal control group; ^#^ significantly different vs. the meleagrin group; ^ significantly different vs. the bleomycin group; ^$^ significantly different vs. the bleomycin + meleagrin group. Differences were considered significant at *p <* 0.05.

**Figure 7:**
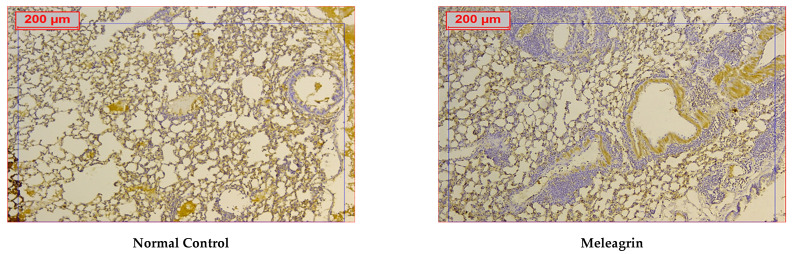

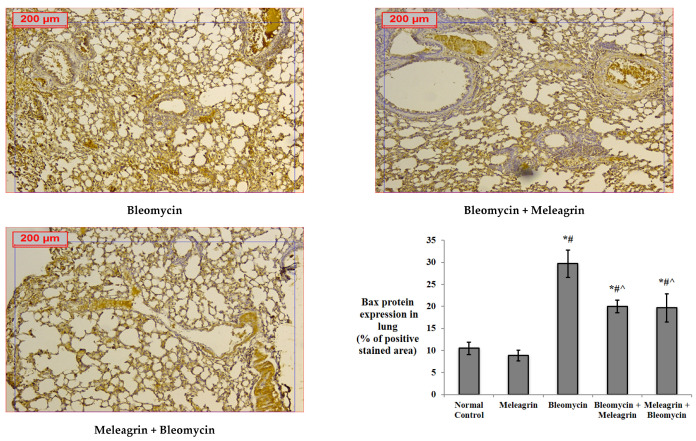
The effect of meleagrin on the protein expression levels of Bax in the lung tissue of the experimental mice determined by immunohistochemistry. Bax = B cell lymphoma-2 associated X protein. Data of the percentage of positive stained area are expressed as mean ± SD and analyzed using one-way ANOVA followed by Bonferroni’s post hoc test (*n* = 8). * significantly different vs. the normal control group; ^#^ significantly different vs. the meleagrin group; ^ significantly different vs. the bleomycin group. Differences were considered significant at *p <* 0.05.

**Figure 8:**
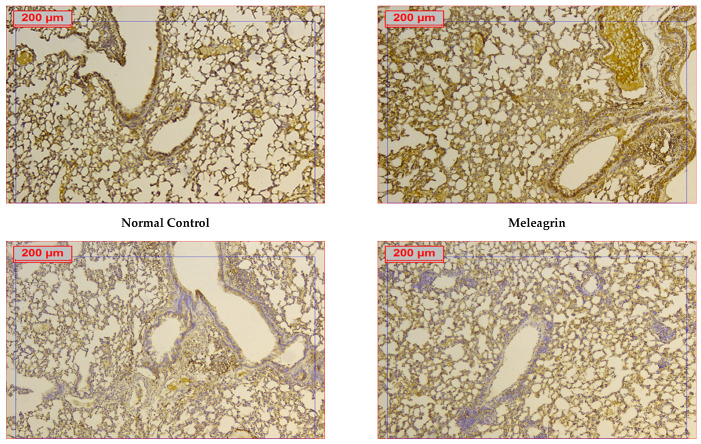

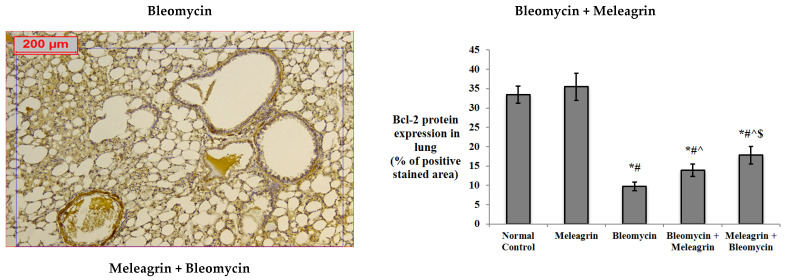
The effect of meleagrin on the protein expression levels of Bcl-2 in the lung tissue of the experimental mice determined by immunohistochemistry. Bcl-2 = B cell lymphoma-2. Data of the percentage of positive stained area are expressed as mean ± SD and analyzed using one-way ANOVA followed by Bonferroni’s post hoc test (*n* = 8). * significantly different vs. the normal control group; ^#^ significantly different vs. the meleagrin group; ^ significantly different vs. the bleomycin group; ^$^ significantly different vs. the bleomycin + meleagrin group. Differences were considered significant at *p <* 0.05.

The expression levels of the apoptotic markers caspase-3 and Bax, and the anti-apoptotic Bcl-2 were assessed in the lung tissue by immunohistochemical staining. The protein expression of all three markers in the group that received meleagrin alone was not significantly different compared to the normal control group (Figure 6, Figure 7 and Figure 8). Bleomycin administration caused a significant increase in the lung expression of caspase-3 (Figure 6) and Bax (Figure 7), along with a significant decline in the expression of Bcl-2 (Figure 8) compared with the normal control group (*p* < 0.05). The use of meleagrin in both groups significantly decreased the percentage of positive stained area in the case of caspase-3 (Figure 6) and Bax (Figure 7), but increased the Bcl-2 percentage of the positive stained area (Figure 8) compared to the bleomycin control group. The use of meleagrin as a protective agent (meleagrin + bleomycin) led to significantly downregulated caspase-3 expression (Figure 6) and significantly upregulated Bcl-2 expression (Figure 8) compared to the bleomycin + meleagrin group (Figure 6).

**Figure 9:**
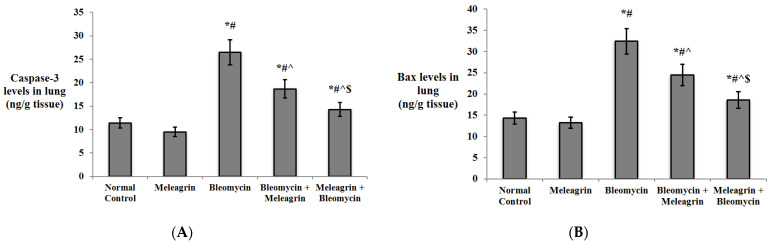

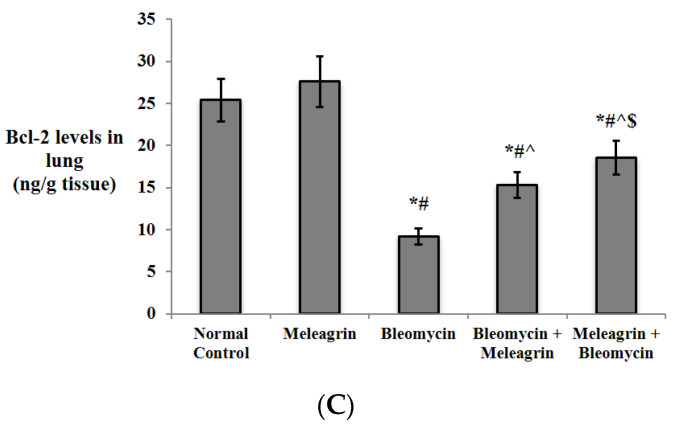
The effect of meleagrin on the protein levels of markers of apoptosis (**A**) caspase-3, **(B)** Bax, and (**C**) Bcl-2 in the lung tissue of the experimental mice. Bcl-2 = B cell lymphoma-2; Bax = Bcl-2 associated x. Data are expressed as mean ± SD and analyzed using one-way ANOVA followed by Bonferroni’s post hoc test (*n* = 8). * significantly different vs. the normal control group; ^#^ significantly different vs. the meleagrin group; ^ significantly different vs. the bleomycin group; ^$^ significantly different vs. the bleomycin + meleagrin group. Differences were considered significant at *p <* 0.05.

Moreover, the protein levels of caspase-3, Bax and Bcl-2 were also determined in the lung tissue of the experimental mice by ELISA (Figure 9). Similar results were obtained, where the administration of meleagrin alone did not significantly affect the levels of all three markers compared to the normal control group, whereas bleomycin administration resulted in a significant increase in the levels of caspase-3 and Bax levels, but a significant decrease in Bcl-2 levels relative to the normal control group (*p* < 0.05). Treatment with meleagrin (bleomycin + meleagrin group) caused a significant decrease in caspase-3 and Bax levels and a significant increase in Bcl-2 levels compared to the bleomycin group. Similarly, the prior administration of meleagrin (meleagrin + bleomycin group) significantly decreased caspase-3 and Bax levels and increased Bcl-2 levels relative to both the bleomycin group and the bleomycin + meleagrin group (Figure 9).

#### 3.2.3. Effect of Meleagrin on the Expression of Fibrotic Markers in the Lung Tissue:

**Figure 10:**
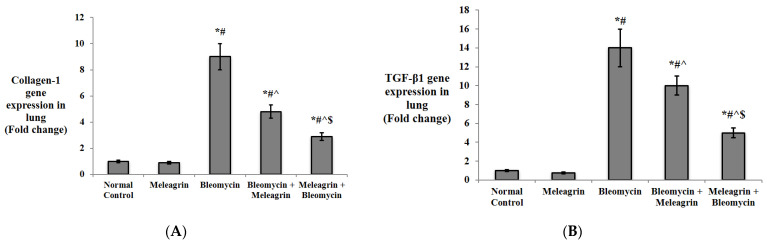

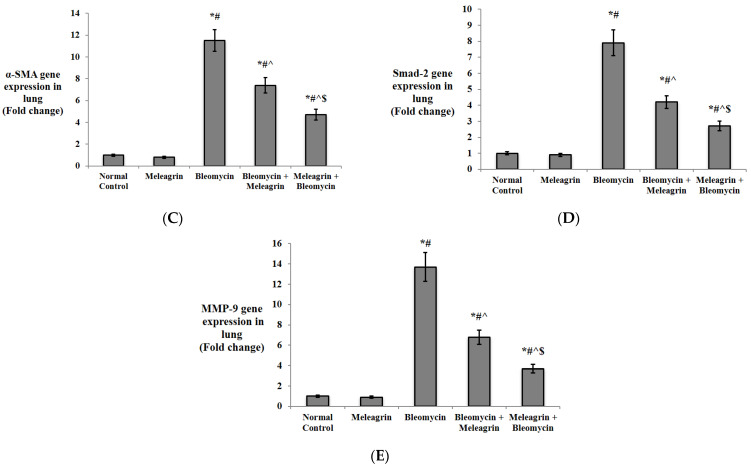
The effect of meleagrin on the gene expression of markers of fibrosis: (**A**) collagen type 1, **(B)** TGF-β1, (**C**) α-SMA, (**D**) Smad-2 and (**E**) MMP-9 in the lung tissue of the experimental mice. TGF-β1 = transforming growth factor-beta 1; α-SMA = alpha smooth muscle actin; MMP-9 = matrix metalloproteinase-9. Data are expressed as mean ± SD and analyzed using one-way ANOVA followed by Bonferroni’s post hoc test (*n* = 8). * significantly different vs. the normal control group; ^#^ significantly different vs. the meleagrin group; ^ significantly different vs. the bleomycin group; ^$^ significantly different vs. the bleomycin + meleagrin group. Differences were considered significant at *p <* 0.05.

Additionally, the gene expression of fibrosis markers in the lung tissue was assessed in the experimental mice (Figure 10). Meleagrin alone did not affect the expression of any of the determined markers. Bleomycin administration significantly upregulated the expression of type I collagen, TGF-β1, α-SMA, Smad-2 and MMP-9 in the lung tissue compared to the normal control group (*p* < 0.05). The anti-fibrotic capability of meleagrin was evidenced by the downregulation of type I collagen, TGF-β1, α-SMA, Smad-2 and MMP-9 expression in the lung tissue in both the treated (bleomycin + meleagrin) and the protected (meleagrin + bleomycin) groups. The current study suggests that the use of meleagrin as a protective agent against bleomycin-induced lung fibrosis was more efficient than its use as a treatment agent. This was emphasized by the significantly lower expression of type I collagen, TGF-β1, α-SMA, Smad-2 and MMP-9 in the lung tissue of the meleagrin + bleomycin group relative to the bleomycin + meleagrin group (Figure 10).

**Figure 11:**
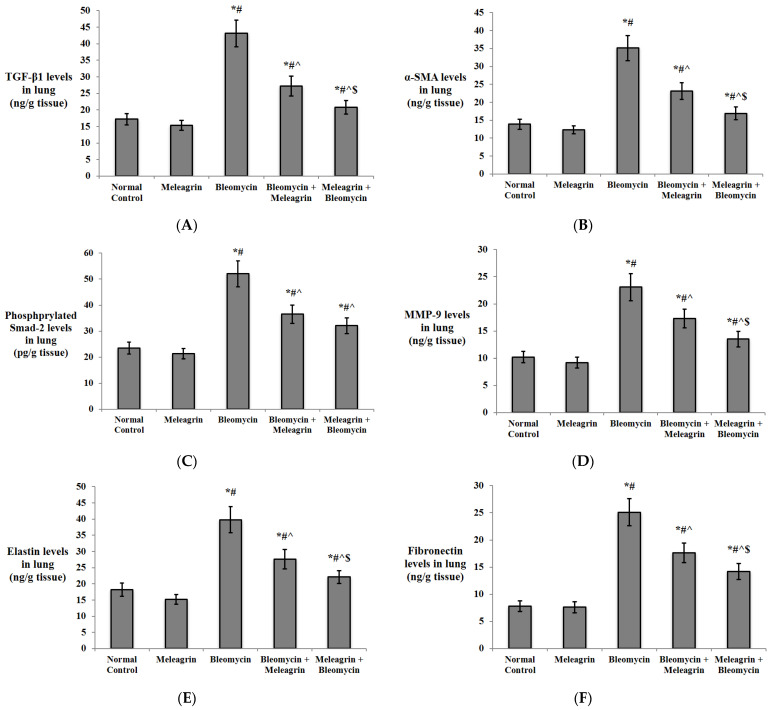
The effect of meleagrin on the protein levels of markers of fibrosis (**A**) TGF-β1, (**B**) α-SMA, (**C**) phosphorylated Smad-2 and (**D**) MMP-9, and the level of extracellular matrix protein (**E**) elastin and (**F**) fibronectin in the lung tissue of the experimental mice. TGF-β1 = transforming growth factor-beta 1; α-SMA = alpha smooth muscle actin; MMP-9 = matrix metalloproteinase-9. Data are expressed as mean ± SD and analyzed using one-way ANOVA followed by Bonferroni’s post hoc test (*n* = 8). * significantly different vs. the normal control group; ^#^ significantly different vs. the meleagrin group; ^ significantly different vs. the bleomycin group; ^$^ significantly different vs. the bleomycin + meleagrin group. Differences were considered significant at *p <* 0.05.

The results of the gene expression of the markers of fibrosis were confirmed by determination of their protein levels in the lung tissue of the experimental mice by ELISA (Figure 11A–D). The levels of TGF-β1, α-SMA, phosphorylated Smad-2 and MMP-9 were all significantly increased in the lung tissue of the mice that received bleomycin alone compared to the normal control and the meleagrin groups (*p* < 0.05). The administration of meleagrin as either a protective or a treatment agent significantly decreased the levels of TGF-β1, α-SMA, phosphorylated Smad-2 and MMP-9 in the lung tissue relative to the bleomycin group. Consistent with their gene expression results, the lung protein levels of TGF-β1, α-SMA and MMP-9 in the meleagrin + bleomycin group were significantly lower than their levels in the bleomycin + meleagrin group (*p* < 0.05) (Figure 11A–D).

Additionally, levels of the extracellular matrix proteins elastin and fibronectin were also determined in the lung tissue of the experimental mice (Figure 11E,F). Both protein levels were not significantly altered in the group that received meleagrin only relative to the normal control group. However, the administration of bleomycin caused a significant increase in the lung levels of elastin and fibronectin compared to the normal control group (*p* < 0.05). Elastin and fibronectin levels were significantly decreased by the administration of meleagrin either before or after bleomycin, with their levels further declined in the meleagrin + bleomycin group compared to the bleomycin + meleagrin group (Figure 11E,F).

#### 3.2.4. Histopathological Examination of the Lung Tissue in the Experimental Mice

**Figure 12:**
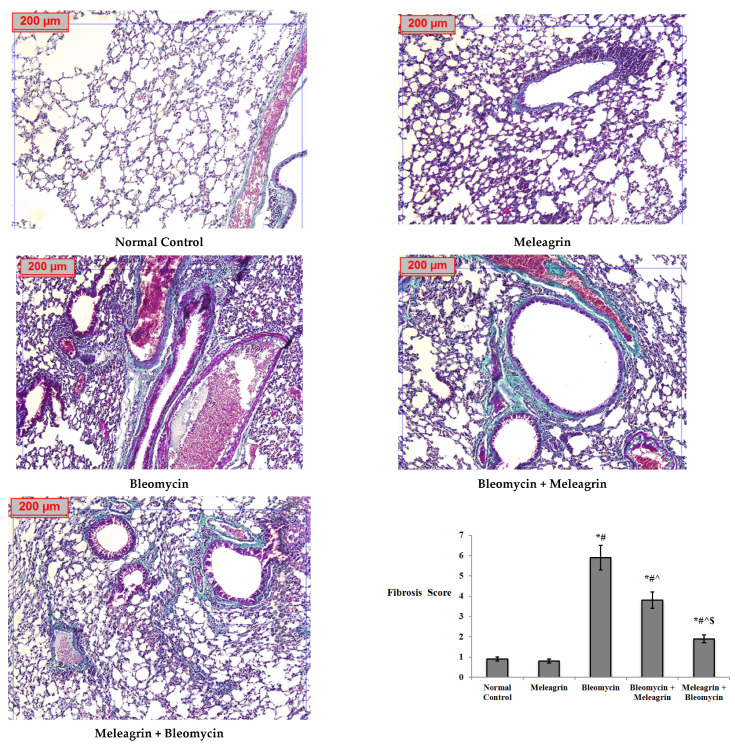
A photomicrography of lung tissue (Masson’s trichrome, ×100). **Normal control group and meleagrin group:** showing normal bronchial and alveolar walls. **Bleomycin group:** showing increased fibrosis with definite damage to lung structure. **Bleomycin + Meleagrin group:** showing moderate thickening of walls with improved lung architecture. **Meleagrin + Bleomycin group:** showing minimal thickening of walls without detectable damage of lung architecture. Fibrosis score is expressed as mean ± SD and analyzed using one-way ANOVA followed by Bonferroni’s post hoc test (*n* = 8). * significantly different vs. the normal control group; ^#^ significantly different vs. the meleagrin group; ^ significantly different vs. the bleomycin group; ^$^ significantly different vs. the bleomycin + meleagrin group. Differences were considered significant at *p <* 0.05.

Consistently, the histopathological examination of the lung tissue using MT staining revealed a normal lung structure and bronchial/alveolar walls in both the normal control and the meleagrin groups (Figure 12). Bleomycin administration caused increased fibrosis with pronounced damage to the lung structure, reflected by the significantly increased lung fibrosis score relative to the normal control group. The administration of meleagrin either as a protective or treatment agent caused a significant decline in the fibrosis score, with the bleomycin + meleagrin group showing a moderate thickening of walls and improved lung architecture, whereas the meleagrin + bleomycin-receiving mice showed better improvement, imaged by a minimal thickening of walls and the lack of detectable damage of the lung architecture (Figure 12).

**Figure 13:**
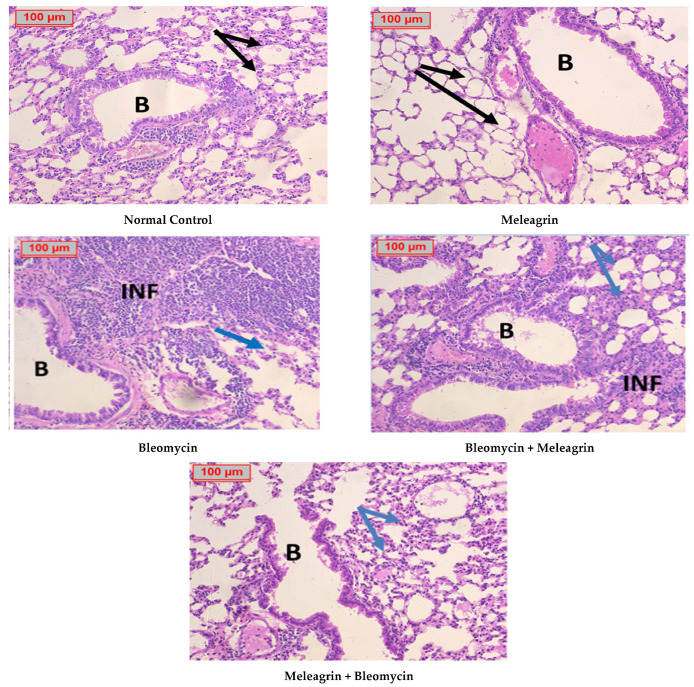
A photomicrography of lung tissue (H&E, ×200). **Normal control group and meleagrin group:** showing normal bronchial tissue as the bronchia have an intact wall and the alveolar walls are also intact with normal thickness (black arrows). **Bleomycin** **group:** showing destructive bronchial tissue mostly infiltrated by massive areas of inflammatory cells with areas of mucin and thickened alveolar walls (blue arrows). **Bleomycin + Meleagrin group:** showing mild improvement with less destructive bronchial tissue infiltrated by focal areas of inflammatory cells, and slightly thickened alveolar walls (blue arrows). **Meleagrin + Bleomycin group:** showing great improvement with no detectable inflammatory cells and almost intact bronchial tissue with minimally thickened alveolar walls. B = bronchia, INF = inflammatory cells.

A histopathological evaluation of the lungs using H&E staining revealed normal bronchial tissue with intact walls in both the normal control group and the meleagrin group. The bleomycin-administered group showed destructive bronchial tissue mostly infiltrated with massive areas of inflammatory cells. Minimal improvement was detected in the treatment group (bleomycin + meleagrin), whereas the protected group (meleagrin + bleomycin) showed great improvement, evidenced by minimizing the destruction of alveolar walls and inflammatory cell infiltration induced by bleomycin (Figure 13).

## 4. Discussion

Pulmonary fibrosis is a chronic progressive interstitial lung disease with an unproven etiology, a poor prognosis and limited therapies. One of the crucial hallmarks of pulmonary fibrosis is the excessive production of extra cellular matrix (ECM) (collagen, fibronectin, elastin, laminin and hyaluronic acid), which is associated with enhanced fibroblasts proliferation and transformation to myofibroblasts [28]. Bleomycin is widely used in rodent models of pulmonary fibrosis. It works by metal ions chelation leading to the formation of pseudoenzymes. The latter react with oxygen to generate superoxide and hydroxyl radicals, eventually triggering DNA strand breaks. Interestingly, the levels of the enzyme bleomycin hydrolase, which inactivates bleomycin, are low in the lung, so it is more susceptible to bleomycin-induced toxicity [5].

In this study, bleomycin was used to induce pulmonary fibrosis by single dose intratracheal administration, leading to inflammatory and fibrotic manifestations. Biochemical and functional changes during the early stages in bleomycin-administered animals have been noted to resemble those observed in human lung fibrosis [29]. It has been demonstrated that bleomycin administration is associated with an initial production of oxidant species, consequently leading to an early inflammatory phase characterized by leukocyte infiltration. Such infiltrated cells synthesize and secrete various cytokines, chemokines and ROS, and the second phase represents fibrosis development [3,29].

Indeed, the redox status and oxidant–antioxidant balance have been previously reported to exert a crucial role in the pathogenesis of lung fibrosis [30]. Consistent with previous studies that have reported an observed decrease in antioxidant levels after bleomycin administration as a result of ROS overproduction [31,32], current results revealed a significant increase in the levels of MDA, an indicator of lipid peroxidation, and, on the contrary, a decrease in GSH content and antioxidant enzymes, SOD and catalase activities in the lung tissue of bleomycin-administered mice.

Nrf2 is a key transcription factor that regulates the cell response to oxidative stress. Under non-stimulus conditions, it is kept bound to Kelch-like ECH-associated protein 1 (Keap1) and is thus retained in the cytoplasm, where it is subjected to Keap1-dependent ubiquitination and proteasomal degradation. Cell exposure to oxidative stress triggers Nrf2 nuclear translocation, binding with antioxidant response elements (ARE) on DNA and the enhanced expression of phase II detoxification enzymes and HO-1 with pronounced antioxidant, anti-inflammatory and anti-apoptotic effects [5]. However, bleomycin-induced oxidative stress triggers the activation of NF-κB signaling and release of inflammatory mediators and cytokines such as TNF-α, IL-1 β, IL-6, IL-8 and IFN-γ by lung cells primarily through TLR4, a pattern recognition receptor, and stimulation. Such events further enhance mitochondrial ROS generation [3,33,34]. Moreover, NF-κB has been previously reported to compete with Nrf2 for its transcriptional co-activator cAMP response element binding protein (CBP) and thus downregulate the transcription and activity of Nrf2 and its downstream target genes [35]. Previous studies have demonstrated decreased Nrf2 and HO-1 expression in lung tissues of bleomycin-administered rats [5,36]. In line with these findings, this study showed that bleomycin administration downregulated the expression of Nrf-2 and decreased the HO-1 level in the lung tissue in comparison to the vehicle control group. In addition, current results revealed upregulated NF-κB, TLR4 and TNF-α expression, along with elevated IL-6 and IFN-γ levels in the lung tissue of the bleomycin group compared to the control group.

As previously reported, the damage and apoptosis of pulmonary tissues is one of the primary pathological features of bleomycin-induced pulmonary fibrosis. Mitochondria have been shown to be involved in the apoptotic process via an altered mitochondrial outer membrane permeability, which is triggered by the oligomerization of pro-apoptotic protein Bax in the mitochondrial outer membrane to form transition pores resulting in the subsequent release of cytochrome and activation of caspases leading to apoptotic cell death. Bcl-2, however, could inhibit cell apoptosis by interfering with Bax activation [37]. In harmony with these findings, this study showed that bleomycin administration caused a significant increase in the immunofluorescence reactivity of caspase-3 and Bax and the percentage of their corresponding positive stained areas, along with a significant decline in those associated with Bcl-2 compared with the normal control group. Moreover, the protein levels of caspase-3, Bax and Bcl-2 were also determined, and similar results were obtained.

A growing body of evidence strongly supports the essential role of elevated cytokines in the development of the second fibrotic phase by virtue of their ability to regulate myofibroblast aggregation and the increased synthesis and accumulation of several ECM components, including collagens, fibronectin and elastin. Such ECM components are known to influence myofibroblast differentiation and participate actively in the fibrotic process in the lung. The overexpression of TNF-α and IL-6 has been previously reported to induce ECM components synthesis and TGF-β expression and stimulate fibroblast proliferation [31,38]. TGF-β1 stimulation enhances the Smad proteins (Smad2 and Smad3) phosphorylation and nuclear translocation, regulating the expression of target genes involved in collagen, fibronectin and elastin synthesis, fibroblasts differentiation, proliferation, transformation and excessive ECM deposition. More to the point, TGF-β1-induced Smad protein phosphorylation has been demonstrated to be associated with an increase in mesenchymal markers production, including α-SMA [39,40,41]. Fibrosis progression has been shown to be attributable to an imbalance between MMPs/TIMPs. Previous studies have reported that the expression of MMPs, endopeptidases that degrade ECM components, is upregulated in bleomycin-induced pulmonary fibrosis, destroying the basement membrane and leading to the invasion of fibroblasts into the alveolar cavity [40,42]. In this study, the gene expression levels of type I collagen, TGF-β1, α-SMA, Smad-2 and MMP-9 were significantly upregulated in the lung tissue of mice with bleomycin-induced pulmonary fibrosis. In addition, the protein levels of TGF-β1, α-SMA, phosphorylated Smad-2 and MMP-9 were all significantly increased in the lung tissue of the mice that received bleomycin alone compared to the normal control and the meleagrin groups (*p* < 0.05), further confirming the results of profibrotic markers gene expression. Moreover, the administration of bleomycin caused a significant increase in the lung levels of the extracellular matrix proteins elastin and fibronectin compared to the normal control group.

Current biochemical investigations were also supported by histopathological evaluation. In agreement with previous reports [40], bleomycin administration triggered inflammatory cell infiltration, fibroblast proliferation, congested blood capillaries and collagen deposition with evident pulmonary fibrosis.

Given the limited availability of effective candidates against pulmonary fibrosis, the identification of innocent anti-fibrotic agents is urgently needed [28]. Recently, due to the high pharmacological bioactivities of indole alkaloids, many studies aimed to explore their anti-fibrotic effect. The use of indole alkaloids has been shown to achieve promising results in the treatment of organ fibrosis. Indole alkaloids and indole derivatives have been reported to alleviate pulmonary, renal, hepatic and myocardial fibrosis through the suppression of inflammation and oxidative stress, and by regulating multiple signaling pathways, including the TGF-β/Smad pathway [43].

The indole alkaloid meleagrin, isolated from Penicillium species, was recently proposed as a promising novel antibiotic agent by virtue of its potential antibacterial activity. In addition, Mady et al. [8] assessed its antitumor potential and showed that meleagrin bears antiproliferative, antimigratory and anti-invasive properties in vitro against a wide panel of c-Met-dependent breast cancer cells. Such in vitro activities were further confirmed in vivo with a significant decrease in the tumor volume and tumor growth by 65% following meleagrin administration without potential systemic toxicity.

To the best of our knowledge, there is no report studying the impact of meleagrin administration against bleomycin-induced pulmonary fibrosis and its possible role on ECM proteins and myofibroblasts proliferation in pulmonary fibrosis. Therefore, the present study aimed to investigate the possible protective effect of meleagrin and elucidate the mechanism of its therapeutic efficacy against bleomycin-induced pulmonary fibrosis by comparing the oxidant–antioxidant, inflammatory, apoptotic and fibrotic status. The data obtained from the study showed the protective effect of meleagrin treatment (5 mg/kg) as evidenced by the reversal of biochemical and histological changes induced by bleomycin.

In the present model, pre- and/or co-treatment with meleagrin boosted the levels of antioxidant enzymes and resulted in lower contents of MDA and higher levels of SOD and catalase activities and GSH content compared to the bleomycin group. Therefore, the protective effects of meleagrin against bleomycin-induced pulmonary fibrosis can be partly attributed to its potent antioxidant activity. Similarly, Zhao et al. [2] have reported that indole alkaloids from *Alstonia scholaris* (L.) R. Br. restored the oxidant–antioxidant balance, protecting against bleomycin-induced pulmonary fibrosis in mice. In addition, it has been reported that indole-6-carboxaldehyde (I6CA), a natural indole derivative from the marine brown algae Sargassum thunbergia, reversed H_2_O_2_-induced cytotoxicity by reducing ROS accumulation, thereby protecting against oxidative stress. Additionally, I6CA significantly promoted Nrf2 expression and enhanced HO-1 activity [44]. Interestingly, various indole-based small molecules have been shown to alleviate organ fibrosis via modulating the Nrf-2/HO-1 cascade [43]. In harmony with these reports, this study demonstrated that the pre- and/or co-administration of meleagrin significantly augmented Nrf2 expression and HO-1 activity in the lung tissue compared to the bleomycin group.

Furthermore, several indole alkaloids have been demonstrated to exert an anti-inflammatory effect. In this regard, decreased TNF-α and TLR4 expressions and inhibited NF-κB activity have been observed with vinpocetine. Additionally, the NF-κB pathway has been shown to be inhibited by indole-3-carbinol (I3C). Another indole alkaloid, isorhynchophylline (isorhy), has been found to alleviate pulmonary inflammation by suppressing IL-1β, TNF-α and IL-6 release [43]. In addition, indole alkaloids from *Alstonia scholaris* have been shown to suppress IL-4 and IFN-γ levels [45]. In line with these findings, current results demonstrated that meleagrin, in both the treatment (bleomycin + meleagrin) and protection (meleagrin + bleomycin) groups, effectively downregulated NF-κB, TLR4 and TNF-α expressions and lowered IL-6 and IFN-γ levels in the lung tissue in comparison with the bleomycin group.

More importantly, it has been previously reported that an indole alkaloid from *Erythrina velutina*, hypaphorine, inhibited the lipopolysaccharide-induced apoptosis of alveolar epithelial cells by abating pro-apoptotic proteins Bax and caspase-3 activities while elevating Bcl2 expression [46]. Consistent with these findings, current results revealed that meleagrin administration in both the treatment (bleomycin + meleagrin) and protection (meleagrin + bleomycin) groups significantly decreased the percentage of the positive stained area in the case of caspase-3 and Bax, but increased the Bcl-2 percentage of the positive stained area compared to the bleomycin group. In line with the immunohistochemistry results, protein caspase-3 and Bax levels, as determined by ELISA, were significantly decreased in both the treatment and protection groups, whereas, on the other hand, protein Bcl-2 levels were significantly increased in comparison to the bleomycin group.

Moreover, decreased expressions of α-SMA and collagen-1, diminished MMPs activity and an inhibited TGF-β1/Smad pathway have been reported to contribute to the anti-fibrotic activity of various indole alkaloids [43]. Rutaecarpine, an indolopyridoquinazoline alkaloid from *E. rutaecarpa*, reduced the expression of several factors, including α-SMA, collagen-I, collagen-III and TGF-β1, attenuating hypoxic-induced right ventricular remodeling in rats [47]. In addition, indirubin, a bis-indole alkaloid from indigo plants or mollusks of the Muricidae family, protected against bleomycin-induced pulmonary fibrosis in mice via decreasing collagen I and α-SMA expressions and inhibiting the TGF-β/Smads signaling pathway [48]. Interestingly, anatabine, a minor tobacco alkaloid, has been shown to reduce the expression of a wide range of factors involved in ECM deposition, including fibronectin and elastin [49]. In harmony with the findings articulated above, current results showed that the anti-fibrotic activity of meleagrin was attributed to the downregulation of type I collagen, TGF-β1, α-SMA, Smad-2 and MMP-9 gene expression in the lung tissue in both the treated (bleomycin + meleagrin) and the protected (meleagrin + bleomycin) groups. Additionally, yjr administration of meleagrin either as a protective or a treatment agent significantly decreased the protein levels of TGF-β1, α-SMA, phosphorylated Smad-2 and MMP-9 in the lung tissue relative to the bleomycin group, with a more pronounced antifibrotic effect in the protection group compared to the treatment group. Elastin and fibronectin levels were significantly decreased by the administration of meleagrin either before or after bleomycin, with their levels further declined in the meleagrin + bleomycin group compared to the bleomycin + meleagrin group. Furthermore, a histological examination revealed that pre- and/or co-treatment with meleagrin improved the pathological signs induced by bleomycin and mitigated alveolitis, congested blood capillaries and collagen deposition. Finally, the use of meleagrin as a protective agent seemed to more efficient against bleomycin -induced lung fibrosis compared to its use as a treatment agent.

Importantly, the administration of meleagrin alone without bleomycin did not elicit any pathologic changes on the lung tissue function and structure, indicating a lack of potential to induce toxic effects.

## 5. Conclusions

In conclusion, meleagrin is an indole alkaloid isolated from deep-sediment-derived *Penicillium chrysogenum* Strain S003. The present study provides evidence that meleagrin exerts a protective effect against bleomycin-induced pulmonary fibrosis in mice. Such a protective effect could be attributable to restoring the oxidant–antioxidant balance through reducing MDA contents and elevating the activities and levels of antioxidant molecules. Importantly, the administration of meleagrin either as a protective or a treatment agent effectively modulated the Nrf-2/HO-1 cascade, downregulated NF-κB, TLR4 and TNF-α gene expressions and lowered IL-6 and IFN-γ levels. In addition, pre- and/or co-treatment with meleagrin inhibited bleomycin-induced cell apoptosis, as evidenced by reducing pro-apoptotic Bax activity with a simultaneous increase in the anti-apoptotic Bcl-2 level leading to diminished caspase-3 activity. Interestingly, the anti-fibrotic activity of meleagrin was attributed to the downregulation of type I collagen, TGF-β1, α-SMA, Smad-2 and MMP-9 gene expression, with a concomitant decrease in the protein levels of TGF-β1, α-SMA, phosphorylated Smad-2, MMP-9, elastin and fibronectin in the lung tissues. Furthermore, its administration amended the histopathological changes induced by bleomycin. In general, the use of meleagrin as a protective agent against bleomycin-induced lung fibrosis was more efficient than its use as a treatment agent. This study supports the implication of meleagrin for future clinical use in lung fibrosis therapy.

## Figures and Tables

**Table 1:** GenBank accession numbers, primer sequences and annealing temperatures of the assessed genes.

GenBank Accession No.	Gene	Primers	Annealing Temperature
NM_009045.5	NF-κB	Forward: 5′-CAATGGCTACACAGGACCA-3′	52 °C
		Reverse: 5′-CACTGTCACCTGGAACCAGA-3′	
NM_013693.3	TNF-α	Forward: 5′-TCTACTGAACTTCGGGGTGATCG-3′	56 °C
		Reverse: 5′-TGATCTGAGTGTGAGGGTCTGGG-3′	
NM_010902.4	Nrf-2	Forward: 5′-CTCTCTGGAGACGGCCATGACT-3′	58 °C
		Reverse: 5′-CTGGGCTGGGGACAGTGGTAGT-3′	
NM_007742.4	Collagen-1	Forward: 5′-GTCCCTGAAGTCAGCTGCATA-3′	53 °C
		Reverse: 5′-TGGGACAGTCCAGTTCTTCAT-3′	
NM_011577.2	TGF-β1	Forward: 5′-CAGTGGCTGAACCAAGGAGAC-3′	53 °C
		Reverse: 5′-ATCCCGTTGATTTCCACGTG-3′	
NM_007392.3	α-SMA	Forward: 5′-ACTGCCGAGCGTGAGATTGT-3′	52 °C
		Reverse: 5′-TGATGCTGTTATAGGTGGTTTCG-3′	
NM_010754.5	Smad-2	Forward: 5′-TAGGTGGGGAAGTGTTTGA-3′	50ᴼC
		Reverse: 5′-TGACAGACTGAGCCAGAAGAGC-3′	
NM_004994.3	MMP-9	Forward: 5′-TCGAAGGCGACCTCAAGTG-3′	53 °C
		Reverse: 5′-TTCGGTGTAGCTTTGGATCCA-3′	
NM_021297.3	TLR-4	Forward: 5′-TTTATTCAGAGCCGTTGGTG-3′	50 °C
		Reverse: 5′- CAGAGGATTGTCCTCCCATT-3′	
NM_007393.5	β-actin	Forward: 5′-ACGGCCAGGTCATCACTATTG-3′	52 °C
		Reverse: 5′-CAAGAAGGAAGGCTGGAAAAGA-3′	

## Data Availability

Data are available within the article.
